# Development and validation of a nomogram for predicting pulmonary infection in patients receiving immunosuppressive drugs

**DOI:** 10.3389/fphar.2023.1255609

**Published:** 2024-01-16

**Authors:** Chuxuan Luo, Yue Zhang, Jiajie Zhang, Chen Jin, Xiaolan Ye, Yan Ren, Huajuan Shen, Maosheng Chen, Yiwen Li, Qiang He, Guangbiao Xu, Lina Shao

**Affiliations:** ^1^ Department of Nephrology, Taizhou Hospital of Zhejiang Province, Wenzhou Medical University, Taizhou, Zhejiang, China; ^2^ Urology and Nephrology Center, Department of Nephrology, Zhejiang Provincial People’s Hospital (Affiliated People’s Hospital, Hangzhou Medical College), Hangzhou, Zhejiang, China; ^3^ Center for General Practice Medicine, Department of Infectious Diseases, Zhejiang Provincial People’s Hospital (Affiliated People’s Hospital, Hangzhou Medical College), Hangzhou, Zhejiang, China; ^4^ Center for Clinical Pharmacy, Cancer Center, Department of Pharmacy, Zhejiang Provincial People’s Hospital (Affiliated People’s Hospital, Hangzhou Medical College), Hangzhou, Zhejiang, China; ^5^ Department of Nephrology, the First Affiliated Hospital of Zhejiang Chinese Medical University (Zhejiang Provincial Hospital of Traditional Chinese Medicine), Hangzhou, Zhejiang, China

**Keywords:** immunosuppressive drugs, LASSO, nomogram, pulmonary infection, predictive model

## Abstract

**Objective:** Pulmonary infection (PI), a severe complication of immunosuppressive therapy, affects patients’ prognosis. As part of this study, we aimed to construct a pulmonary infection prediction (PIP) model and validate it in patients receiving immunosuppressive drugs (ISDs).

**Methods:** Totally, 7,977 patients being treated with ISDs were randomised 7:3 to the developing (*n* = 5,583) versus validation datasets (*n* = 2,394). Our predictive nomogram was established using the least absolute shrinkage and selection operator (LASSO) and multivariate COX regression analyses. With the use of the concordance index (C-index) and calibration curve, the prediction performance of the final model was evaluated.

**Results:** Among the patients taking immunosuppressive medication, PI was observed in 548 (6.9%). The median time of PI occurrence after immunosuppressive therapy was 123.0 (interquartile range: 63.0, 436.0) days. Thirteen statistically significant independent predictors (sex, age, hypertension, DM, malignant tumour, use of biologics, use of CNIs, use of methylprednisolone at 500 mg, use of methylprednisolone at 40 mg, use of methylprednisolone at 40 mg total dose, use of oral glucocorticoids, albumin level, and haemoglobin level) were screened using the LASSO algorithm and multivariate COX regression analysis. The PIP model built on these features performed reasonably well, with the developing C-index of 0.87 (sensitivity: 85.4%; specificity: 81.0%) and validation C-indices of 0.837, 0.829, 0.832 and 0.830 for predicting 90-, 180-, 270- and 360-day PI probability, respectively. The decision curve analysis (DCA) and calibration curves displayed excellent clinical utility and calibration performance of the nomogram.

**Conclusion:** The PIP model presented herein could aid in the prediction of PI risk in individual patients who receive immunosuppressive treatment and help personalise clinical decision-making.

## 1 Introduction

Immunosuppressive drugs (ISDs) are a class of drugs that exert immunosuppressive effects through various mechanisms and are primarily used to clinically modulate the immune response of patients (Suthanthiran et al., 1996; Barshes et al., 2004; Allison, 2005). Since the establishment of their immunosuppressive action, ISDs have been widely recommended as first-line therapeutics in organ transplantation cases and the treatment of autoimmune disorders (Kasiske et al., 2010; Radhakrishnan and Cattran, 2012; Fanouriakis et al., 2019). With the widespread application of glucocorticoids and other immunosuppressants, an increasing number of latent adverse effects is linked with this class of agents in recent years, and this limits their use (Penfornis and Kury-Paulin, 2006; Htwe and Khardori, 2017; Chotiyarnwong and McCloskey, 2020). Pulmonary infection (PI) is a common comorbidity in immunosuppressed patients and contributes to an exceptionally high mortality rate (Godbole and Gant, 2013; Ahuja and Kanne, 2014), which it has various potential reasons. One reason could be that immunosuppressants inhibit the immune function of patients, which significantly increases their susceptibility to a variety of microbial pathogens and the incidence of infection, especially PI, directly threatening the lives of many patients (Stahn and Buttgereit, 2008). Moreover, the symptoms of infection in patients could be overshadowed by the long-term course of immunosuppressants, which makes it difficult for early diagnosis and treatment, thus rendering rapid progression and a poor prognosis (Poowuttikul et al., 2019). The results of a 10-year cohort study from China revealed that patients with pneumonia who previously received active immunosuppressant therapy had a greater risk of mortality when hospitalised (Yin et al., 2021). Therefore, it is crucial to understand the risk factors and identify patients at a high risk of PI when initiating immunosuppressant therapy. However, there is a lack of visual prediction models that can be applied in broad and large populations.

The nomogram model, as a multi-factor calibrated visualisation tool, has been extensively used to predict various outcomes in clinical practice, and it can provide the rationale for clinicians to develop more effective and individualised therapy regimens (Iasonos et al., 2008). Accordingly, this research was devised to establish a simple and effective nomogram prediction model for PI and validate it in patients receiving immunosuppressive therapy.

## 2 Materials and methods

### 2.1 Definitions

The diagnostic criteria for PI in the current research encompassed the following elements: 1) Clinical manifestations comprised the onset of a new cough or expectoration, or the exacerbation of existing respiratory tract symptoms, accompanied by or without purulent sputum, chest pain, dyspnea, hemoptysis, fever, and rales detected during lung auscultation; 2) Leukocyte count exceeding 10 × 10^9^/L or falling below 4 × 10^9^/L (Kalil et al., 2016); 3) Imaging characteristics encompassed the emergence of infiltration, consolidation, ground-glass opacity, or effusion observed in chest plain films or computed tomography scans (Wunderink et al., 1992; Koenig and Truwit, 2006). Patients who satisfied the third criterion, in conjunction with either the first or second criterion, were subjected to a clinical diagnosis of PI. Diagnosis of PI was reviewed and reconfirmed by an experienced specialist in infectious diseases (JZ).

Immunosuppressive agents used in this study were calcineurin inhibitors (CNIs) (tacrolimus and cyclosporine A), cyclophosphamide (CTX), mycophenolate mofetil (MMF), biologics (rituximab, infliximab, etanercept, adalimumab, tocilizumab, abatacept and bortezomib), azathioprine, methotrexate, leflunomide, Tripterygium wilfordii, hydroxychloroquine, and glucocorticoids.

### 2.2 Eligibility criteria

The inclusion criteria were as follows: 1) patients using ISDs according to electronic medical records; 2) a follow-up period of longer than 14 days after initiating immunosuppressive therapy.

The exclusion criteria were as follows: 1) age less than 18 years old; 2) PI occurred within 14 days of immunosuppressive therapy; 3) patients with pre-existing PI who need to receive ISDs treatment, such as acute exacerbations of chronic obstructive pulmonary disease (AECOPD) and COVID-19.

### 2.3 Data sources and processing

**FIGURE 1:**
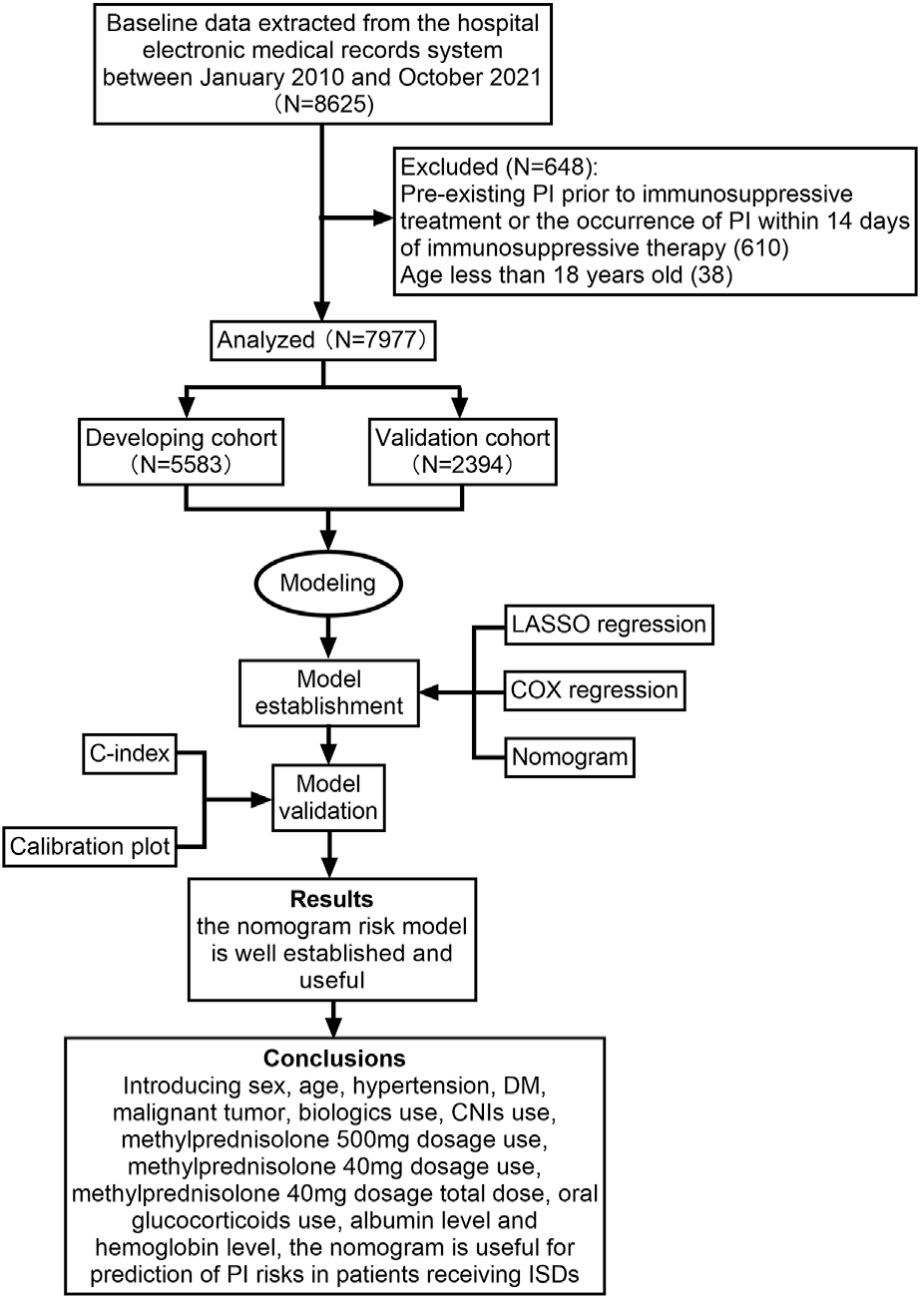
Flow diagram of the study design. CNIs, calcineurin inhibitors; DM, diabetes mellitus; ISDs, immunosuppressive drugs; LASSO, least absolute shrinkage and selection operator; PI, pulmonary infection.

Demographic characteristics and laboratory data of, and physician’s orders for these patients including, but not limited to, sex, age, hypertension, diabetes mellitus (DM), malignant tumour, use of immunosuppressive agents, albumin level, haemoglobin level and lymphocyte level were excerpted from the hospital’s electronic patient management system. The study design flowchart is depicted in Figure 1.

### 2.4 Statement of human rights and ethics

Keeping in conformity with the Helsinki Declaration (as revised in 2013), our study received ethical permission from the Zhejiang Provincial People’s Hospital Ethics Committee (No. 2021QT345) (World Medical Association, 2013).

### 2.5 Informed consent

Informed consent from the patients was waived as the study was retrospective in nature.

### 2.6 Statistical analyses

The baseline characteristics of the two cohorts are summarised as frequency count (percentage) for count variables and median (interquartile range [IQR]) for metric variables. To measure between-group differences, count variables were subjected to chi-square or Fisher’s exact test, and metric variables were subjected to t-test or Wilcoxon test, depending on the distribution.

Imputation was conducted if missing values were <20%. The LASSO method was adopted to screen significant covariates and prevent the overfitting of the model. In this regression model, the absolute size of the coefficients of a regression model is penalised according to the value of λ, which is a LASSO regularisation parameter. In the presence of larger penalties, the estimates of weaker factors shrink toward zero, resulting in only the strongest factors remaining in the model. The most predictive covariates were chosen based on the minimum of λ (λmin). Thereafter, we incorporated the variables identified via LASSO regression analysis into COX regression models, and the variables that were consistently statistically significant were chosen to construct the nomogram.

A nomogram was constructed based on the final model. To evaluate the predictive performance in terms of discrimination and calibration, validation of the derived nomogram was carried out. An assessment of discrimination was performed using the receiver operating characteristic (ROC) curve and concordance index (C-index). A comparison of the observed PI rates with predictions from the final model was used to evaluate calibration. R software, version 4.1.0 (2021-05-18; R Foundation for Statistical Computing, Vienna, Austria) was employed for the statistical analyses presented. R packages including mice, VIM, missForest, survival, survminer, rms, glmnet, regplot, survivalROC, and survcomp were utilised in the current study. A two-tailed P-value of <0.05 was set to denote statistical significance for all tests.

## 3 Results

### 3.1 Baseline characteristics of the developing and validation datasets

In total, 8,625 patients using ISDs between January 2010 and October 2021 were consecutively selected for this study. Among these patients, 610 were excluded because of PI before immunosuppressive treatment or the occurrence of PI within 14 days of immunosuppressive therapy, and 38 patients were excluded as they were under 18 years of age. Ultimately, 7,977 patients were selected for the current analysis. All participants were randomly assigned to either the developing dataset (*n* = 5,583) or validation dataset (*n* = 2,394) at a 7:3 ratio. The detailed study flow was given in Figure 1. There were no statistically significant differences in the demographic factors between the sets (Table 1). The prevalence of PI was 6.9% (384/5,583) in the developing set and 6.9% (164/2,394) in the validation set. The median time of PI after immunosuppressive therapy was 123.0 (IQR: 63.0, 436.0) days.

**TABLE 1:** Demographics and baseline characteristics of the study cohort.

	Overall	Developing cohort	Validation cohort	*P*
n	7977	5583	2394	
Follow up time (day)	134.0 (64.0, 495.0)	136.0 (64.0, 501.50)	133.0 (64.0, 469.0)	0.407
Pulmonary infection n(%)	548 (6.9)	384 (6.9)	164 (6.9)	1
Pulmonary infection time (day)	123.0 (63.0, 436.0)	125.0 (63.0, 438.5)	121.0 (63.0, 433.0)	0.476
Sex (female) n(%)	5431 (68.1)	3830 (68.6)	1601 (66.9)	0.136
Age (years)	55 (44, 66)	56 (44, 66)	55 (43, 66)	0.207
Smoking habit n(%)	986 (12.4)	685 (12.3)	301 (12.6)	0.733
Drinking habit n(%)	676 (8.5)	483 (8.7)	193 (8.1)	0.411
Hypertension n(%)	1357 (17.0)	949 (17.0)	408 (17.0)	0.987
Diabetes mellitus n(%)	628 (7.9)	432 (7.7)	196 (8.2)	0.524
Malignant tumor n(%)	2709 (34.0)	1899 (34.0)	810 (33.8)	0.897
Organ transplanation n(%)	172 (2.2)	111 (2.0)	61 (2.5)	0.135
Autoimmune disease n(%)	5056 (63.4)	3546 (63.5)	1510 (63.1)	0.728
Kidney disease n(%)	1676 (21.0)	1146 (20.5)	530 (22.1)	0.112
CTX n(%)	3178 (39.8)	2208 (39.5)	970 (40.5)	0.432
CTX total dose (mg)	3800(2700, 5200)	3800(2700, 5100)	3800 (2800, 5400)	0.378
MMF n(%)	1047 (13.1)	737 (13.2)	310 (12.9)	0.788
MMF duration (day)	140 (48, 533)	146 (47, 548)	140 (49, 471)	0.709
CNIs n(%)	1339 (16.8)	947 (17.0)	392 (16.4)	0.541
CNIs duration (day)	153 (48, 408)	150 (50, 420)	161 (45, 395)	0.486
Biologics n(%)	454 (5.7)	315 (5.6)	139 (5.8)	0.813
Azathioprine n(%)	1825 (22.9)	1309 (23.4)	516 (21.6)	0.07
Azathioprine duration (day)	159 (34, 680)	163 (35, 646)	154 (32, 767)	0.079
Methotrexate n(%)	1203 (15.1)	853 (15.3)	350 (14.6)	0.472
Methotrexate duration (day)	119 (28, 525)	119 (28, 506)	109 (28, 591)	0.451
Leflunomide n(%)	1559 (19.5)	1072 (19.2)	487 (20.3)	0.251
Leflunomide duration (day)	182 (49, 727)	177 (50, 700)	193 (40, 784)	0.236
Tripterygium wilfordii n(%)	2166 (27.2)	1550 (27.8)	616 (25.7)	0.065
TW duration (day)	170 (44, 567)	175 (44, 575)	162 (43, 549)	0.065
Hydroxychloroquine n(%)	1825 (22.9)	1309 (23.4)	516 (21.6)	0.07
Hydroxychloroquine duration (day)	159 (34, 680)	163(35, 646)	154 (32, 767)	0.079
Pred 500 mg n(%)	166 (2.1)	108 (1.9)	58 (2.4)	0.189
Pred 500 mg total dose (mg)	2000 (1500, 2803)	2000 (1500, 2500)	2000 (1500, 3000)	0.159
Pred 40 mg n(%)	1081 (13.6)	739 (13.2)	342 (14.3)	0.223
Pred 40 mg total dose (mg)	640 (280, 1280)	600 (280, 1280)	650 (280, 1283)	0.199
Oral glucocorticoids n(%)	2743 (34.4)	1908 (34.2)	835 (34.9)	0.561
Oral glucocorticoids duration (day)	131 (34, 405)	133 (33, 412)	126 (35, 393)	0.634
SMZ n(%)	626 (7.8)	434 (7.8)	192 (8.0)	0.742
Albumin, g/L	40.2 (36.0, 43.5)	40.3 (36.2, 43.5)	40.1 (35.8, 43.5)	0.575
Globulin, g/L	29.0 (25.8, 32.5)	29.0 (25.8, 32.5)	29.0 (25.9, 32.3)	0.942
Creatinine, μmol/L	71.4 (62.9, 84.9)	71.2 (63.0, 84.8)	71.9 (62.7, 85.0)	0.559
Uric acid, umol/L	295 (238, 366)	295 (238, 366)	297 (238, 366)	0.957
Fasting blood glucose, mmol/L	5.03 (4.63, 5.56)	5.02 (4.63, 5.56)	5.04 (4.62, 5.57)	0.494
Phosphorus, mmol/L	1.18 (1.05, 1.31)	1.18 (1.04, 1.31)	1.18 (1.05, 1.31)	0.267
Monocyte %	5.70 (4.40, 7.20)	5.60 (4.40, 7.10)	5.70 (4.40, 7.20)	0.926
Monocyte, ×10⁹ /L	0.34 (0.26, 0.48)	0.34 (0.26, 0.48)	0.34 (0.25, 0.48)	0.841
Basophil %	0.30 (0.10, 0.40)	0.20 (0.10, 0.40)	0.30 (0.10, 0.40)	0.365
Basophil	0.02 (0.01, 0.03)	0.02 (0.01, 0.03)	0.02 (0.01, 0.03)	0.656
Eosinophil %	1.20 (0.50, 2.40)	1.20 (0.50, 2.30)	1.30 (0.50, 2.40)	0.262
Eosinophil, ×10⁹ /L	0.07 (0.03, 0.14)	0.07 (0.03, 0.14)	0.07 (0.03, 0.14)	0.136
Lymphocyte %	28.1 (21.1, 35.0)	28.1 (21.0, 34.9)	28.0 (21.3, 35.1)	0.488
Lymphocyte count, ×10⁹ /L	1.67 (1.22, 2.20)	1.67 (1.22, 2.20)	1.67 (1.22, 2.20)	0.69
Neutrophil to lymphocyte ratio	2.24 (1.60, 3.33)	2.24 (1.60, 3.35)	2.25 (1.58, 3.28)	0.344
Platelet to lymphocyte ratio	125.8 (90.7, 172.7)	125.9 (90.7, 172.1)	125.4 (90.7, 174.2)	0.992
White blood cell count, ×10⁹/L	6.11 (4.78, 7.87)	6.10 (4.78, 7.88)	6.14 (4.77, 7.85)	0.793
Hemoglobin, g/L	128.0 (114.0, 139.0)	128.0 (115.0, 139.0)	128.0 (114.0, 139.0)	0.899
Platelets, ×10⁹ /L	214.0 (167.0, 264.0)	214.0 (166.0, 264.0)	214.0 (169.0, 265.0)	0.507

CTX, cyclophosphamide; MMF, mycophenolate mofetil; CNIs, calcineurin inhibitors; TW, Tripterygium wilfordii; Pred, methylprednisolone; SMZ, sulfamethoxazole.

### 3.2 Predictor selection

**FIGURE 2:**
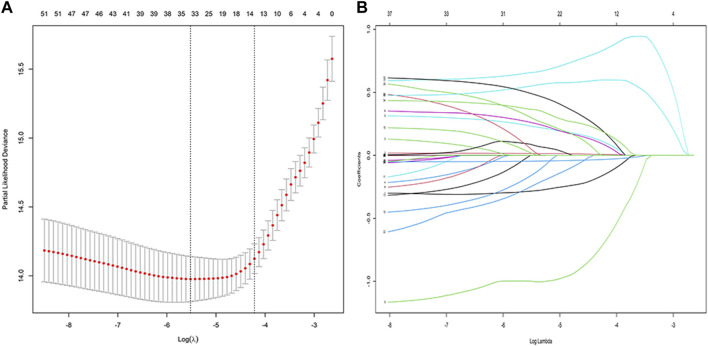
Variable selection using the least absolute shrinkage and selection operator (LASSO) regression model: **(A)** LASSO coefficient profiles of the 58 baseline features; **(B)** Tuning parameter (λ) selection in the LASSO model used 10-fold cross-testing via minimum criteria.

**FIGURE 3:**
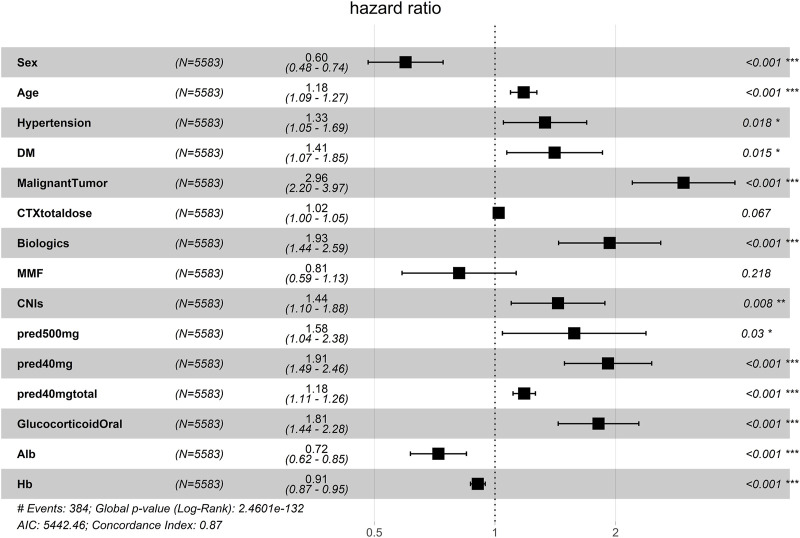
Multivariate COX regression analysis of the developing cohort. Alb, albumin; CNIs, calcineurin inhibitors; CTX, cyclophosphamide; DM, diabetes mellitus; Hb, haemoglobin; MMF, mycophenolate mofetil; pred, methylprednisolone.

A total of 53 general variables measured at admission to the hospital were included in the LASSO regression analysis. Participants’ characteristics were shown in Supplementary Table S1. Based on the LASSO regression analysis results, 15 features were chosen to be potential predictors of PI, including sex, age, hypertension, DM, malignant tumour, CTX total dose, use of biologics, use of MMF, use of CNIs, use of methylprednisolone at 500 mg, use of methylprednisolone at 40 mg, total cumulative dose of methylprednisolone at 40 mg, use of oral glucocorticoids, albumin level, and haemoglobin level. The screening for the LASSO analysis is shown in Figure 2. The significant features from LASSO were subsequently chosen for further multivariate COX regression analysis. Thirteen variables were finally screened as statistically significant independent predictors of PI. The results of the multivariate COX regression analysis are presented in Figure 3.

### 3.3 Nomogram construction

**FIGURE 4:**
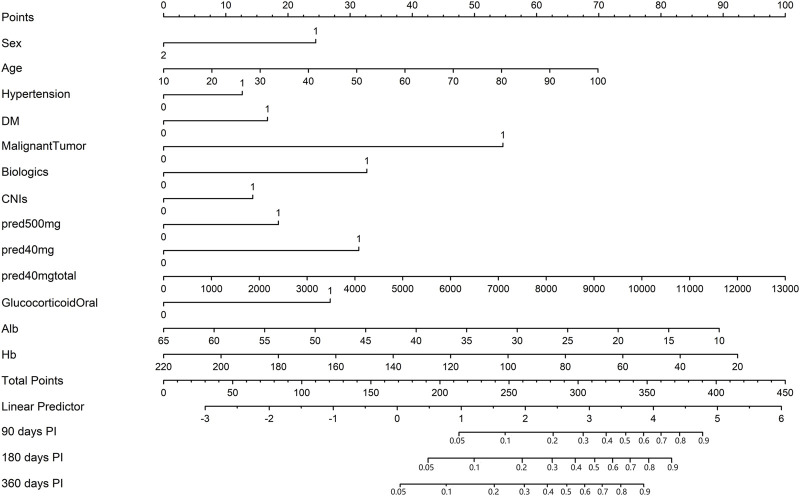
The nomogram of the pulmonary infection prediction (PIP) model. In the nomogram, each patient’s value is displayed on an axis for each variable, and a line is drawn upward to determine the number of points each variable receives. On the Total Points axis is the sum of these numbers, and a line is drawn downward to the survival axes to determine 90-, 180- and 360-day probability of PI. Alb, albumin; CNIs, calcineurin inhibitors; DM, diabetes mellitus; Hb, haemoglobin; PI, pulmonary infection; pred, methylprednisolone.

Thirteen of the clinical parameters (sex, age, hypertension, DM, malignant tumour, use of biologics, use of CNIs, use of methylprednisolone at 500 mg, use of methylprednisolone at 40 mg, use of methylprednisolone at 40 mg total dose, use of oral glucocorticoids, albumin level, and haemoglobin level) were integrated to construct a pulmonary infection prediction (PIP) model in patients receiving ISDs (Figure 4).

Notably, patients with the following features presented a greater probability of developing PI: male, old age, hypertension, DM, malignant tumour, use of biologics, use of CNIs, use of methylprednisolone at 500 mg, use of methylprednisolone at 40 mg, high-dose methylprednisolone, use of oral glucocorticoids, and low albumin and haemoglobin levels.

Moreover, the longer the length of the line, the greater the effect of these factors on the risk of developing PI. As found from the nomogram, the use of methylprednisolone at 40 mg total dose had the greatest effect on the occurrence of PI, whereas the presence of hypertension was observed to have the least effect. The top line of the nomogram corresponded to the score for each factor. Scores for each of these parameters were pooled, with higher scores indicating a higher risk of developing PI.

### 3.4 Nomogram validation

**FIGURE 5:**
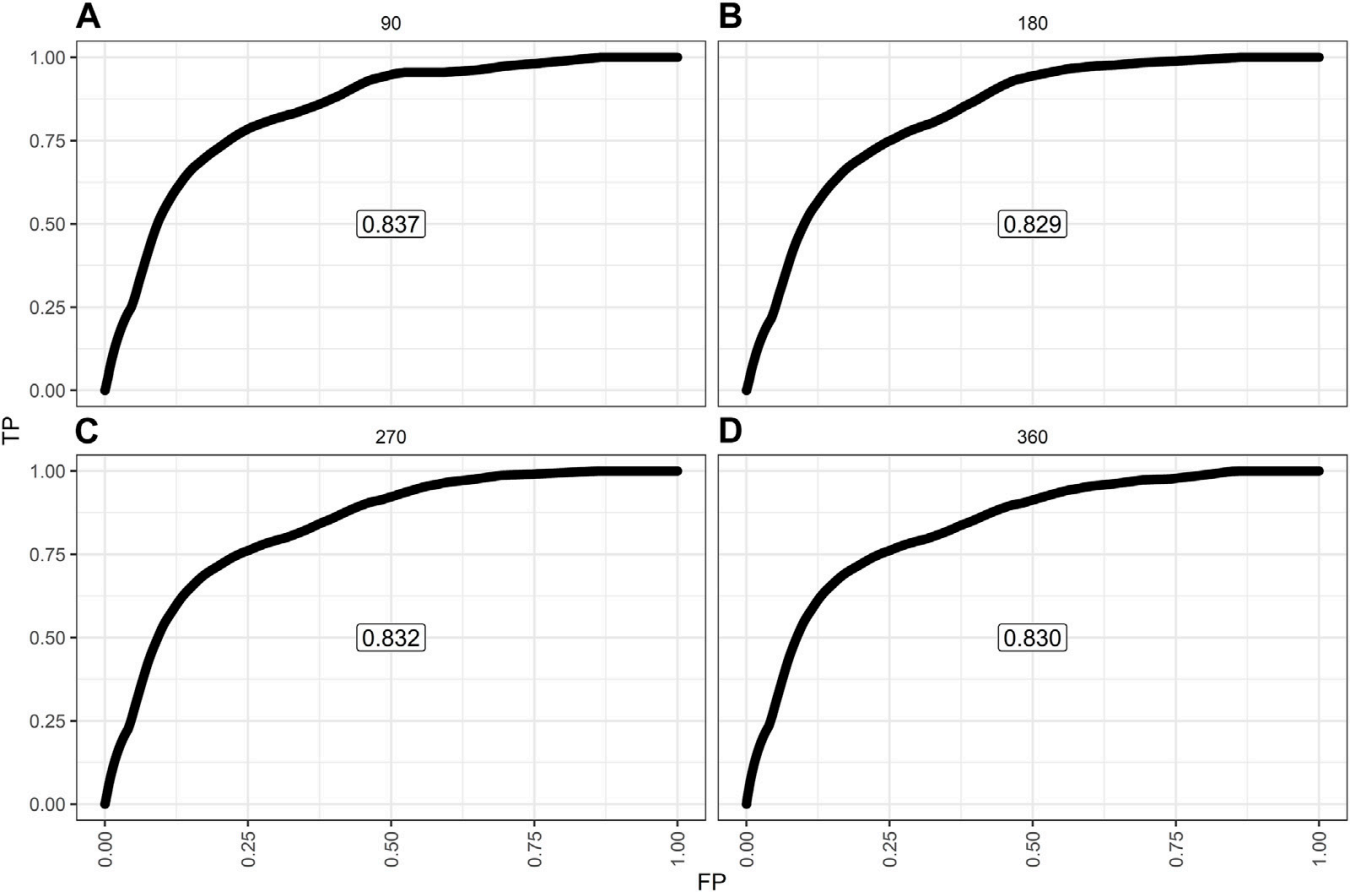
The receiver operating characteristic (ROC) curves based on the nomogram in the validation dataset: **(A)** ROC curve predicting 90-day probability of pulmonary infection (PI); **(B)** ROC curve predicting 180-day probability of PI; **(C)** ROC curve predicting 270-day probability of PI; **(D)** ROC curve predicting 360-day probability of PI.

**FIGURE 6:**
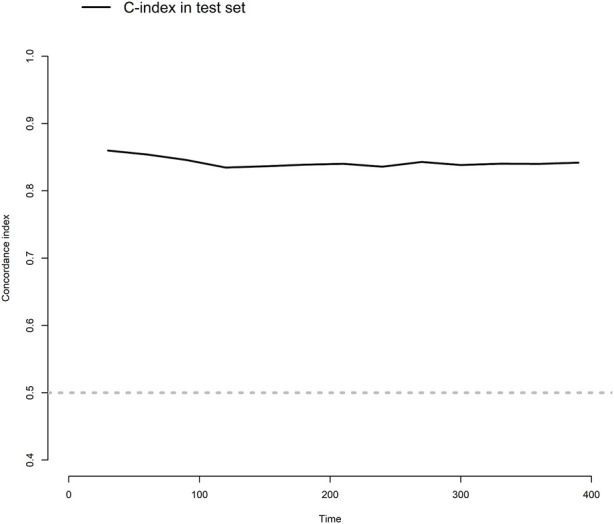
The dynamic alterations of concordance index (C-index) for the model in the validation dataset.

**FIGURE 7:**
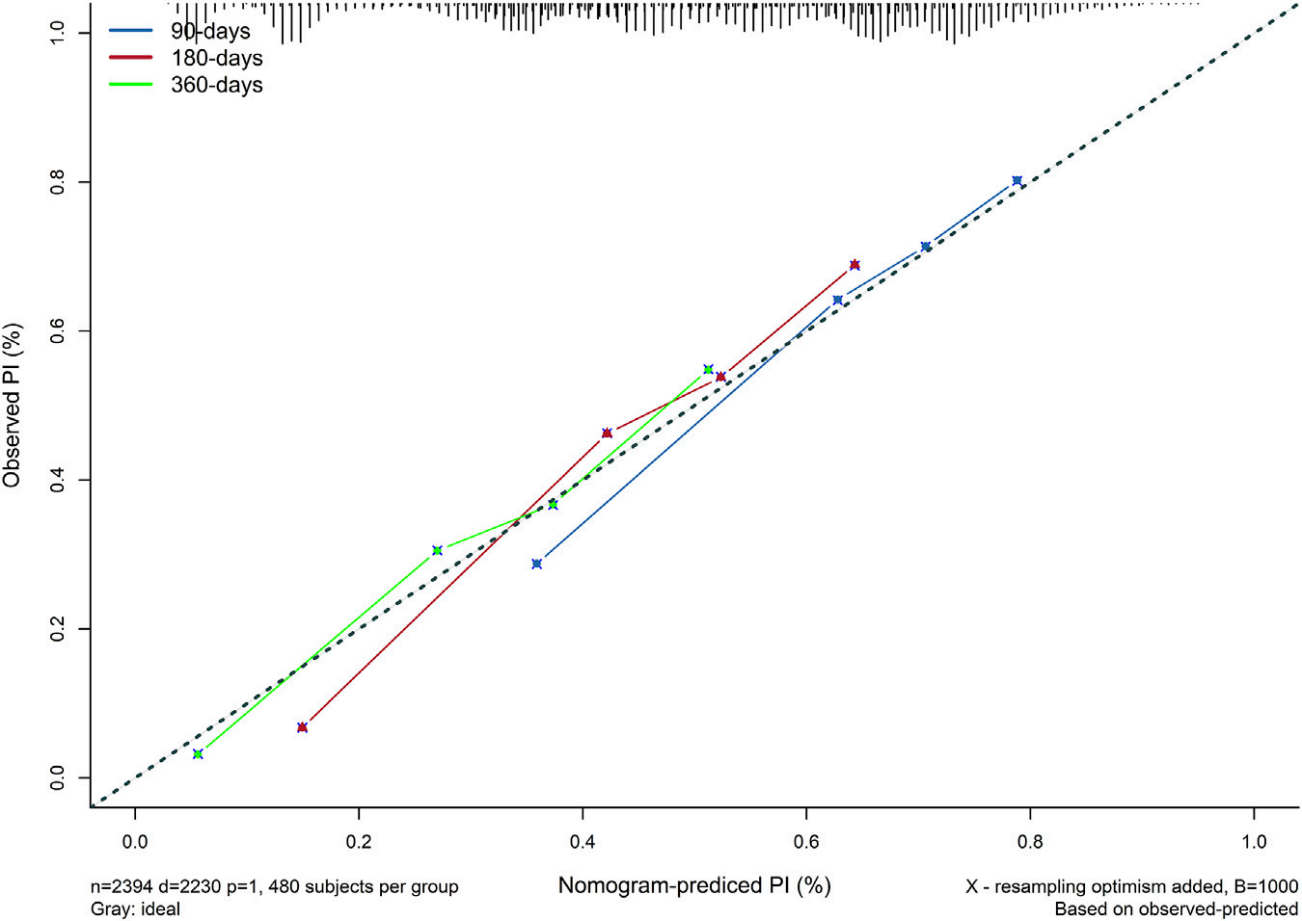
The calibration diagram of the model in the validation dataset.

**FIGURE 8:**
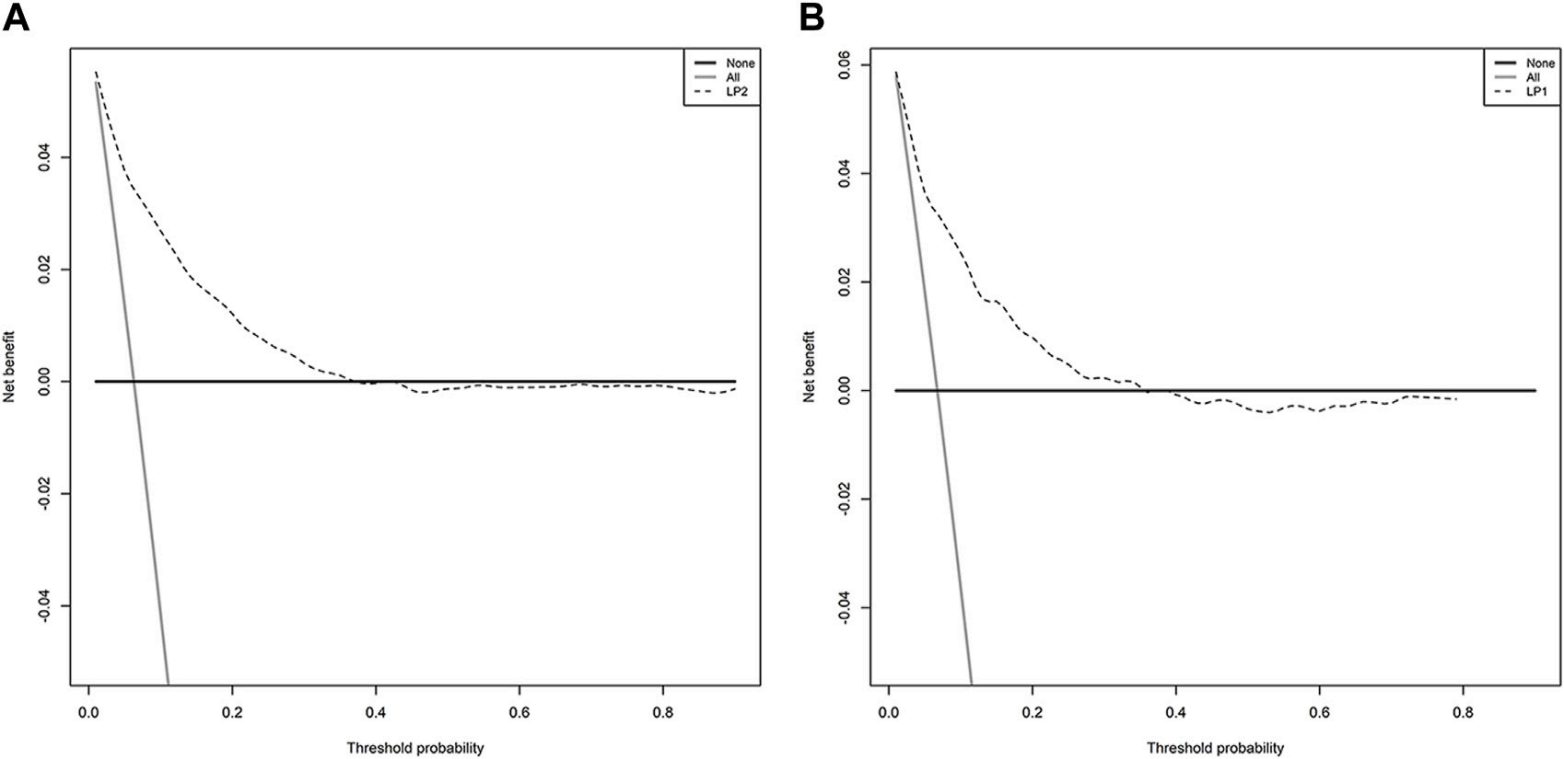
The decision curve analysis (DCA) of the model: **(A)** DCA in the developing dataset; **(B)** DCA in the validation dataset.

The model showed a high degree of discrimination, with the developing C-index of 0.87 and the validation C-indices of 0.837, 0.829, 0.832 and 0.830 for predicting 90-, 180-, 270- and 360-day PI probability, respectively (Figure 5). The dynamic alterations of the C-indices for the PIP model in the validation cohort are shown in Figure 6. The calibration plot also displayed excellent concordance between the predicted probability of PI and observations, which indicated good calibration of the model in the validation dataset (Figure 7). The decision curve analysis (DCA) of the developing and validation datasets proved the potential clinical value of the model (Figure 8).

## 4 Discussion

We built a simplified prediction model based on 13 easily accessible variables selected using LASSO and multivariate regression analyses to facilitate the personalised estimation of the likelihood of PI and validated it in patients who received immunosuppressant medications. The LASSO regression analysis allows the shrinkage of the coefficients of the less contributive variables to be exactly zero, which effectively deals with the multicollinearity problem in the model and the enormous number of clinical factors (Hepp et al., 2016). The nomogram provides a simple pictorial representation of sophisticated mathematical calculations, and it has been recognised as a reliable and valuable predictive tool for clinical use (Xie et al., 2015). However, it must be clarified that the relatively large number of parameters involved in this model did not affect its usefulness and feasibility. This is because the combination of two or three ISDs is the most commonly used therapy, and it is seldom that all of the listed ISDs are simultaneously prescribed in clinical practice. Further, all patients receiving immunosuppressant therapy were included in our study, regardless of hospital departments. As a result, our predictive model seemed to have a better generalizability to some extent. In 2019, Wang et al. (2019), utilising clinical data of 333 patients, proposed using the AIM-7C score for the early evaluation of the risk of developing PI after the initiation of a cyclosporine regimen. Nevertheless, this score was only applicable to patients with primary membranous nephropathy, and not to all patients receiving immunosuppressive therapy. To our understanding, this is the inaugural instance of a visual nomogram model designed explicitly for the early detection of PI in all patients treated with ISDs.

In this study, we found that the prevalence of PI was 6.9% (548/7,977), illustrating that patients receiving immunosuppressive therapy are a high-risk population for PI. Furthermore, the results showed that the incidence rate of PI among males was 11.9% (302/2,546), whereas that among females was 4.2% (246/5,431), demonstrating that male patients were more vulnerable to PI than females. The reason for this phenomenon could not be elucidated. One possible reason is the different effects of sex hormones on the immune response. Previous studies have reported that testosterone has an immunosuppressive effect, whereas oestrogens tend to reinforce immunological hyper-response (Beery, 2003). Additionally, the majority of males have a history of smoking and alcohol consumption (Fujii et al., 2021; Shrestha et al., 2021). Smoking and alcohol consumption can aggravate underlying diseases and reduce cardiopulmonary function, which is another possible reason for this observation. Another independent risk factor that we found was the age of the patients. There is increasing evidence that the lung immune system declines with age, increasing the risk of developing infections and chronic inflammatory diseases, as well as the mortality rate (Larbi et al., 2008). Moreover, we found low albumin and haemoglobin levels to be independent risk factors for PI development, and this was consistent with the findings of previous research (Levy et al., 2005; Wang et al., 2019). As a well-established marker of malnutritional status, a low serum albumin level strongly suggests poor dietary choices in patients. Long-term immunosuppressive therapy can further affect nutrient balance, which in turn reduces the body’s immunity to pathogens. Patients receiving immunosuppressive therapy who have anaemia may experience weakened immune systems, further increasing their risk of infection. Furthermore, we found that malignant tumours, and the use of biologics and CNIs, were significantly associated with the occurrence of PI. It is generally known that patients with malignant tumours often experience complications with infection owing to their immunocompromised status (Azevedo et al., 2020). Infection is frequently cited as an adverse effect in the administration of biologics and CNIs (Thaunat et al., 2004; Evens et al., 2011; Kelesidis et al., 2011; Sułkowska et al., 2012; High and Olivry, 2020). The pathogenic microorganisms identified in patients with infection included viruses, bacteria, fungi, and virus-bacteria co-infection. Besides the above clinical parameters, we found that the use of methylprednisolone at 500 mg, use of methylprednisolone at 40 mg, total cumulative dose of methylprednisolone at 40 mg, and use of oral glucocorticoids were independent predictors of PI development. Long-term use of glucocorticoids can inhibit the antigen-antibody reaction in the human body, thereby leading to the development of infection (McEwen, 2008). A previous animal study confirmed the relationship of glucocorticoid use with the development of PI (Reis e Sousa, 2001). As such, rational clinical use of glucocorticoids appears to be particularly valuable. In addition, DM and hypertension were correlated with an elevated risk of developing PI. Indeed, DM has been considered a significant risk factor for lower respiratory tract infections in susceptible patients (Klekotka et al., 2015). Chronic hypertension damages blood vessels, resulting in pulmonary oedema, pulmonary congestion, systemic hypoxia, and even PI (Ponte et al., 2013).

Based on 13 independent risk factors (male, age, hypertension, DM, malignant tumour, use of biologics, use of CNIs, use of methylprednisolone at 500 mg, use of methylprednisolone at 40 mg, total cumulative dose of methylprednisolone at 40 mg, use of oral glucocorticoids, and low albumin and haemoglobin levels) established from a large cohort with sufficient sample size, we constructed the first practical nomogram model and demonstrated that it was advantageous for the individualised risk stratification of PI in patients taking immunosuppressants. Our nomogram model demonstrated satisfactory prediction ability, with C-indices of 0.837, 0.829, 0.832, and 0.830 for the 90-, 180-, 270- and 360-day probability of PI in the validation set, respectively, which demonstrated the model’s favourable discriminative ability to differentiate patients who were at a risk of developing PI from those who were not. Furthermore, the calibration plot demonstrated a strong agreement between the calibration and standard curves in the validation cohort, suggesting that the predicted occurrence of PI was close to the observed data. In addition to acknowledging uncontrolled factors, such as sex, age, and malignant tumours, medical staff should strengthen the management of controllable factors. In this prediction model, we consider a value greater than 0.5 as high risk and a value less than 0.5 as low risk. If the value is greater than 0.5, we will not only increase the frequency and content of follow-up, but also communicate with the patient about the risk of infection and adjust the treatment plan if necessary. Once high-risk cases are detected, aggressive preventive interventions should also be undertaken at the earliest opportunity to reduce the morbidity rate of PI. For instance, it is imperative to strongly advocate smoking cessation for all patients receiving immunosuppressants who are still smoking. And nursing staff should assume the responsibility of instructing patients in respiratory function exercises to enhance lung function. Moreover, regular nutritional screening and management should be reinforced as a fundamental element of preventive measures, ensuring efficient support in maintaining optimal patient health.

A number of limitations were identified in the current study. In the first place, due to its retrospective nature, its generalizability was limited by the fact that it was restricted to a single site. Yet, our study enrolled all patients treated with ISDs in a single center irrespective of disciplines, which might somewhat compensate for the aforementioned drawback. Second, our nomogram has not yet undergone external validation, and thus, the extrapolation of our nomogram to other cohorts remains unknown. Further independent external validation using additional large datasets to verify these findings has been planned. Third, some patients were administered more than one ISD during the study, which unavoidably introduced bias into the study. Consequently, further studies should take into account the potential interactions among multiple drugs. Last but not least, our study did not grade the risk of developing PI in patients with immunosuppressive therapy. Accordingly, we plan to explore this issue in our future research.

## 5 Conclusion

To summarize, sex, age, hypertension, DM, malignant tumour, use of biologics, use of CNIs, use of methylprednisolone at 500 mg, use of methylprednisolone at 40 mg, total cumulative dose of methylprednisolone at 40 mg, use of oral glucocorticoids, and albumin and haemoglobin levels were independent predictors of the occurrence of PI in patients who were receiving immunosuppressant therapy. The nomogram model established in this study has the potential to assist in the development of an optimal therapeutic intervention for this condition, thereby effectively reducing the occurrence of PI. This suggests that our nomogram model holds promise for widespread implementation in clinical practice.

## Data Availability

The raw data supporting the conclusion of this article will be made available by the authors, without undue reservation.

## Abbreviations

C-index, concordance index; CNIs, calcineurin inhibitors; CTX, cyclophosphamide; DCA, decision curve analysis; DM, diabetes mellitus; IQR, interquartile range; ISDs, immunosuppressive drugs; λmin, minimum of λ; LASSO, least absolute shrinkage and selection operator; MMF, mycophenolate mofetil; PI, pulmonary infection; PIP, pulmonary infection prediction; ROC; receiver operating characteristic.
