# Supplementary material for: Development and validation of a nomogram for predicting pulmonary infection in patients receiving immunosuppressive drugs
Source: Front Pharmacol. 2024 Jan 16;14:1255609. doi: 10.3389/fphar.2023.1255609 (PMC10825965; doi:10.3389/fphar.2023.1255609)
Supplement: Supplementary file 1 [file Table1.docx]

Supplementary Material

# Supplementary Table

**Supplementary Table S1.** Baseline characteristics of study patients with pulmonary infection

|  |  | **Overall** | **Non pulmonary infection** | **Pulmonary infection** | **P** |
| --- | --- | --- | --- | --- | --- |
| n |  | 5583 | 5199 | 384 |  |
| Sex n(%) | Male | 1753 (31.4) | 1537 (29.6) | 216 (56.2) | <0.001 |
|  | Female | 3830 (68.6) | 3662 (70.4) | 168 (43.8) |  |
| Age (years) (median [IQR]) |  | 56 [44, 66] | 55 [44, 65] | 64 [49, 72] | <0.001 |
| Smoking habit n(%) | No | 4898 (87.7) | 4602 (88.5) | 296 (77.1) | <0.001 |
|  | Yes | 685 (12.3) | 597 (11.5) | 88 (22.9) |  |
| Drinking habit n(%) | No | 5100 (91.3) | 4768 (91.7) | 332 (86.5) | 0.001 |
|  | Yes | 483 (8.7) | 431 (8.3) | 52 (13.5) |  |
| Hypertension n(%) | No | 4634 (83.0) | 4382 (84.3) | 252 (65.6) | <0.001 |
|  | Yes | 949 (17.0) | 817 (15.7) | 132 (34.4) |  |
| Diabetes mellitus n(%) | No | 5151 (92.3) | 4841 (93.1) | 310 (80.7) | <0.001 |
|  | Yes | 432 (7.7) | 358 (6.9) | 74 (19.3) |  |
| Malignant tumor n(%) | No | 3684 (66.0) | 3490 (67.1) | 194 (50.5) | <0.001 |
|  | Yes | 1899 (34.0) | 1709 (32.9) | 190 (49.5) |  |
| Organ transplanation n(%) | No | 5472 (98.0) | 5109 (98.3) | 363 (94.5) | <0.001 |
|  | Yes | 111 (2.0) | 90 (1.7) | 21 (5.5) |  |
| Autoimmune disease n(%) | No | 2037 (36.5) | 1816 (34.9) | 221 (57.6) | <0.001 |
|  | Yes | 3546 (63.5) | 3383 (65.1) | 163 (42.4) |  |
| Kidney disease n(%) | No | 4437 (79.5) | 4106 (79.0) | 331 (86.2) | 0.001 |
|  | Yes | 1146 (20.5) | 1093 (21.0) | 53 (13.8) |  |
| CTX n(%) | No | 3375 (60.5) | 3226 (62.1) | 149 (38.8) | <0.001 |
|  | Yes | 2208 (39.5) | 1973 (37.9) | 235 (61.2) |  |
| CTX total dose (mg) |  | 3680 [2475,4800] | 3680 [2475,4800] | 7200 [4400,9900] | <0.001 |
| MMF n(%) | No | 4846 (86.8) | 4554 (87.6) | 292 (76.0) | <0.001 |
|  | Yes | 737 (13.2) | 645 (12.4) | 92 (24.0) |  |
| MMF duration (day) |  | 145.9 [47.4, 548.5] | 136.9 [46.6, 537.3] | 212.6 [48.9, 627.6] | 0.154 |
| CNIs n(%) | No | 4636 (83.0) | 4381 (84.3) | 255 (66.4) | <0.001 |
|  | Yes | 947 (17.0) | 818 (15.7) | 129 (33.6) |  |
| CNIs duration (day) |  | 150.0 [49.9, 419.9] | 143.4 [46.9, 386.3] | 219.0 [77.9, 631.0] | 0.001 |
| Biologics n(%) | No | 5268 (94.4) | 4987 (95.9) | 281 (73.2) | <0.001 |
|  | Yes | 315 (5.6) | 212 (4.1) | 103 (26.8) |  |
| Azathioprine n(%) | No | 4274 (76.6) | 3950 (76.0) | 324 (84.4) | <0.001 |
|  | Yes | 1309 (23.4) | 1249 (24.0) | 60 (15.6) |  |
| Azathioprine duration (day) |  | 163.0 [35.1, 645.9] | 155.9 [33.2, 618.8] | 345.4 [90.3, 1215.4] | 0.003 |
| Methotrexate n(%) | No | 4730 (84.7) | 4437 (85.3) | 293 (76.3) | <0.001 |
|  | Yes | 853 (15.3) | 762 (14.7) | 91 (23.7) |  |
| Methotrexate duration (day) |  | 119.0 [28.1, 506.0] | 141.6 [31.9, 575.7] | 37.7 [19.1, 160.3] | <0.001 |
| Leflunomide n(%) | No | 4511 (80.8) | 4153 (79.9) | 358 (93.2) | <0.001 |
|  | Yes | 1072 (19.2) | 1046 (20.1) | 26 (6.8) |  |
| Leflunomide duration (day) |  | 176.6 [49.8, 699.9] | 176.6 [50.1, 697.8] | 171.1 [43.7, 1477.6] | 0.904 |
| Tripterygium wilfordii n(%) | No | 4033 (72.2) | 3727 (71.7) | 306 (79.7) | 0.001 |
|  | Yes | 1550 (27.8) | 1472 (28.3) | 78 (20.3) |  |
| TW duration (day) |  | 175.0 [43.5, 575.5] | 166.5 [42.0, 552.9] | 443.9 [130.7, 1057.8] | <0.001 |
| Hydroxychloroquine n(%) | No | 4274 (76.6) | 3950 (76.0) | 324 (84.4) | <0.001 |
|  | Yes | 1309 (23.4) | 1249 (24.0) | 60 (15.6) |  |
| Hydroxychloroquine duration (day) |  | 163.0 [35.1, 645.9] | 155.9 [33.2, 618.8] | 345.4 [90.3, 1215.4] | 0.004 |
| Pred 500mg n(%) | No | 5475 (98.1) | 5120 (98.5) | 355 (92.4) | <0.001 |
|  | Yes | 108 (1.9) | 79 (1.5) | 29 (7.6) |  |
| Pred 500mg total dose (mg) |  | 2000 [1500, 2500] | 2000 [1500, 3200] | 1500 [1100, 2200] | 0.073 |
| Pred 40mg n(%) | No | 4844 (86.8) | 4647 (89.4) | 197 (51.3) | <0.001 |
|  | Yes | 739 (13.2) | 552 (10.6) | 187 (48.7) |  |
| Pred 40mg total dose (mg) |  | 600 [280,1280] | 555 [280, 1040] | 1120 [360, 2660] | <0.001 |
| Oral glucocorticoids n(%) | No | 3675 (65.8) | 3540 (68.1) | 135 (35.2) | <0.001 |
|  | Yes | 1908 (34.2) | 1659 (31.9) | 249 (64.8) |  |
| Oral glucocorticoids duration (day) |  | 133.0 [33.2, 412.1] | 125.9 [30.0, 392.8] | 199.7 [78.8, 505.5] | <0.001 |
| SMZ n(%) | No | 5149 (92.2) | 4863 (93.5) | 286 (74.5) | <0.001 |
|  | Yes | 434 (7.8) | 336 (6.5) | 98 (25.5) |  |
| Albumin, g/L |  | 40.3 [36.2, 43.5] | 40.5 [36.6, 43.7] | 36.1 [31.2, 39.9] | <0.001 |
| Globulin, g/L |  | 29.0 [25.8, 32.5] | 29.1 [25.9, 32.5] | 27.9 [23.9, 31.6] | <0.001 |
| Creatinine, μmol/L |  | 71.2 [63.0, 84.8] | 70.8 [62.9, 84.0] | 78.5 [66.3, 96.2] | <0.001 |
| Uric acid , umol/L |  | 295 [238, 366] | 293 [236, 362] | 327 [265, 416] | <0.001 |
| Fasting blood glucose, mmol/L |  | 5.02 [4.63, 5.56] | 5.02 [4.64, 5.54] | 5.04 [4.50, 5.76] | 0.824 |
| Phosphorus, mmol/L |  | 1.18 [1.04, 1.31] | 1.17 [1.04, 1.31] | 1.20 [1.04, 1.36] | 0.044 |
| Monocyte % |  | 5.60 [4.40, 7.10] | 5.60 [4.40, 7.00] | 6.10 [4.50, 8.22] | <0.001 |
| Monocyte,×10⁹ /L |  | 0.34 [0.26, 0.48] | 0.34 [0.26, 0.47] | 0.38 [0.24, 0.56] | 0.016 |
| Basophil % |  | 0.20 [0.10, 0.40] | 0.30 [0.10, 0.40] | 0.20 [0.00, 0.40] | <0.001 |
| Basophil,×10⁹ /L |  | 0.02 [0.01, 0.03] | 0.02 [0.01, 0.03] | 0.01 [0.00, 0.02] | <0.001 |
| Eosinophil % |  | 1.20 [0.50, 2.30] | 1.20 [0.50, 2.35] | 1.00 [0.20, 2.20] | 0.001 |
| Eosinophil,×10⁹ /L |  | 0.07 [0.03, 0.14] | 0.07 [0.03, 0.14] | 0.06 [0.01, 0.14] | <0.001 |
| Lymphocyte % |  | 28.1 [21.0, 34.9] | 28.2 [21.3, 35.0] | 25.4 [17.9, 34.8] | 0.001 |
| Lymphocyte count, ×10⁹ /L |  | 1.67 [1.22, 2.20] | 1.70 [1.24, 2.20] | 1.40 [0.97, 2.02] | <0.001 |
| Neutrophil to lymphocyte ratio |  | 2.24 [1.60, 3.35] | 2.22 [1.60, 3.31] | 2.50 [1.59, 4.02] | 0.01 |
| Platelet to lymphocyte ratio |  | 125.9 [90.6, 172.] | 126.0 [91.1, 171.1] | 123.1 [77.8, 193.1] | 0.567 |
| White blood cell count, ×10⁹ /L |  | 6.10 [4.78, 7.88] | 6.11 [4.82, 7.82] | 5.99 [4.33, 8.81] | 0.724 |
| Hemoglobin, g/L |  | 128 [115, 139] | 128 [116, 139] | 117 [97, 131] | <0.001 |
| Platelets,×10⁹ /L |  | 214 [166, 264] | 215 [168, 264] | 190 [126, 251] | <0.001 |

CNIs, calcineurin inhibitors; CTX, cyclophosphamide; IQR, interquartile range; MMF, mycophenolate mofetil; Pred, methylprednisolone; SMZ, sulfamethoxazole; TW, Tripterygium wilfordii.
